# Effects of an orally supplemented probiotic on the autophagy protein LC3 and Beclin1 in placentas undergoing spontaneous delivery during normal pregnancy

**DOI:** 10.1186/s12884-020-02905-z

**Published:** 2020-04-15

**Authors:** Ping Yang, Zhe Li, Kian Deng Tye, Yuyi Chen, Tong Lu, Zonglin He, Juan Zhou, Xiaomin Xiao

**Affiliations:** 1 Department of Obstetrics and Gynecology, The First Affiliated Hospital of Jinan University, Guangzhou, China; 2 Department of Obstetrics and Gynecology, The Third Affiliated Hospital of Sun Yat-Sen University, Guangzhou, China; 3 Department of Otolaryngology, Shenzhen Long Hua District Central Hospital, Shenzhen, China; 4 Faculty of Medicine, International School, Jinan University, Guangzhou, China

**Keywords:** Probiotic, Autophagy, Normal pregnancy, Spontaneous delivery

## Abstract

**Background:**

Probiotic supplementation has been shown to be beneficial and is now widely promoted as an auxiliary medicine for maternal health, but the underlying mechanism is still unclear. Thus, this study aimed to explore the effects of probiotic supplementation on the placental autophagy-related proteins LC3 and Beclin1.

**Method:**

A population-based cohort of specimens was collected under sterile conditions from 37 healthy nulliparous pregnant women who underwent systemic examination and delivered at the First Affiliated Hospital of Jinan University (Guangzhou, China). At 32 weeks of gestation, the pregnant women in the probiotic group were orally supplemented with golden bifid, and the pregnant women in the control group received no probiotic. Pregnant women with pregnancy-associated complications were excluded in the follow-up period, and 25 pregnant women undergoing spontaneous delivery were enrolled. The placental tissue specimens were collected at term. Western blotting was used to detect the protein expression, and qRT-PCR was used to detect the mRNA expression of the placental autophagy-related proteins LC3 and Beclin1.

**Results:**

①There was no significant difference in the expression levels of either LC3 or Beclin1 protein between the two groups (*P* > 0.05). ②Probiotic supplementation induced a modest but not significant decrease in the content of LC3-mRNA with a significant decrease in the content of Beclin1-mRNA (*P* < 0.05).

**Conclusion:**

Our study indicates that probiotic supplementation may reduce Beclin1-mRNA levels.

## Background

Probiotic foods are ubiquitous in our daily lives, and the number of people taking probiotics is increasing. Pregnant women in particular, attach great importance to nutritional supplements during pregnancy. Therefore, many pregnant women voluntarily take certain probiotics supplements, such as golden bifid and acidophilus milk, during pregnancy. The use of probiotics in the United States and Canada ranges from 1.3 to 3.6%, whereas the probability of using probiotics in pregnant women in the Netherlands has risen to 13.7% [1]. The International Scientific Association for Probiotics and Prebiotics (ISAPP) defines probiotics as live microorganisms that can induce a health benefit on the host if administered in adequate amounts. Generally, Lactobacillus and Bifidobacterium are widely used [2]. However, metabolic byproducts, dead microorganisms or other nonviable products based on microorganisms are not probiotics. Currently, golden bifid (i.e., *live combined Bifidobacterium and Lactobacillus tablets*) is voluntarily purchased and taken by pregnant women as a nonprescription drug. Moreover, recent research has shown that probiotics serve to mediate certain biological effects, including diminishing free radical injury, inflammation, and tumor progression and regulating lipid metabolism [3–6].

Autophagy is an evolutionary adaptive response of eukaryotic cells that moves senescent cells and organelles to lysosomes for degradation and recycling and ultimately maintains homeostasis of the intracellular environment [7, 8]. The placenta, a unique organ formed during pregnancy, is an important survival organ for material exchange between the fetus and mother. It can provide nutrition, gas exchange, and immune and metabolic support for developing fetuses. Studies have shown that the basal level of placental trophoblast autophagy plays an important role in the whole pregnancy process of embryo implantation and placental vascular recasting [9, 10]. If autophagy is excessive, it will change from a protective mechanism to a damaging mechanism, hindering the metabolic functioning of placental trophoblasts and leading to certain placental-derived diseases, such as preeclampsia and fetal growth restriction [11, 12]. Moreover, LC3 (microtubule associated protein l light chain3) and Beclin1 are commonly used to detect autophagy and further reflect the metabolic function of the placenta [13, 14]. Recent studies have suggested that probiotics can mildly regulate macrophages to enhance the immune response to foreign invaders, the mechanism underlying which is the enhancement of macrophage autophagy to defend against infection by probiotics [15]. Lactobacillus acidophilus and *Bacillus clausii* are potent activators of innate immune responses in the murine macrophage cell line RAW264.7 [16]. Probiotics mediate the immunostimulatory activity by interacting with both microorganism-associated molecular patterns and Toll-like receptors, which also participate in the stimulation of autophagy in macrophages. Despite this evidence, only a few studies have explored the regulation of autophagy by probiotics [17–19]. In view of the scarcity of information regarding the role of probiotics in mediating autophagy in humans, especially the effects of probiotics on placental autophagy in pregnant women during pregnancy, the present study aimed to investigate the changes caused by orally supplemented probiotics in the expression of the autophagy-related proteins LC3 and Beclin1 that reflect the metabolic functions of the placenta.

In this study, the pregnant women in the probiotic group voluntarily took a golden bifid supplement at 32 weeks of gestation. The aim of this research was to investigate the effect of probiotics on placental autophagy and to provide novel insight into probiotic-mediated placental protection.

## Methods

### Participants

**Fig. 1:**
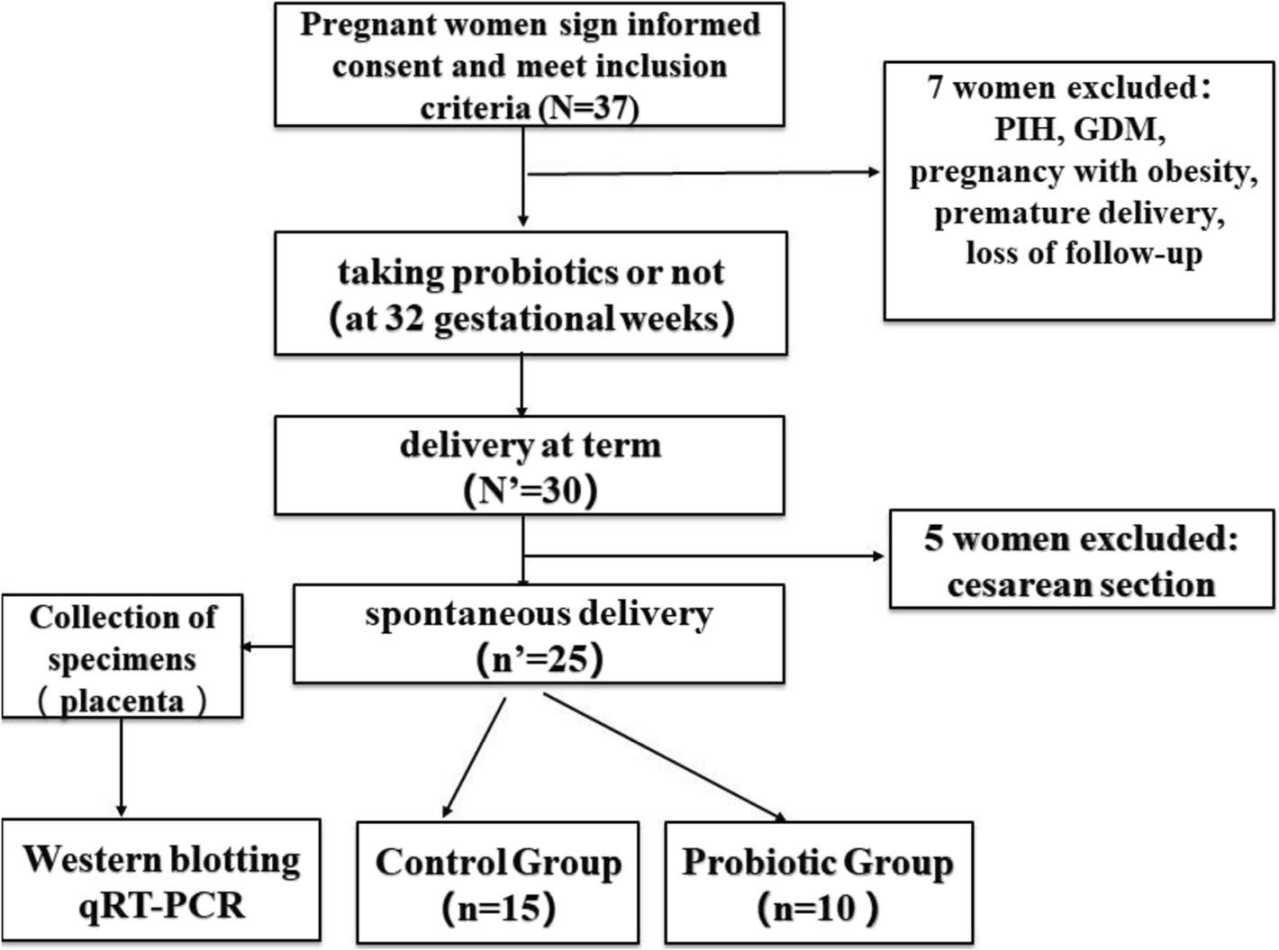
Technical flow chart for screening research subjects

According to the principle of informed consent, 37 patients with normal physiological pregnancy who planned to give birth in the First Affiliated Hospital of Jinan University (Guangzhou, Guangdong, China) and met the inclusion criteria were recruited. At 32 weeks of gestation, the pregnant women in the probiotic group received a golden bifid supplement (i.e., *live combined Bifidobacterium and Lactobacillus tablets*) orally twice a day until the full term of pregnancy was reached (standard: 0.5 g/tablet, each tablet contained 0.5*10^7^/CFU of live *Bifidobacterium* and 0.5*10^6^/CFU of *Lactobacillus* and *Streptococcus*). The pregnant women in the control group did not receive any probiotics. During the study follow-up period, seven subjects with perinatal complications were excluded (including 1 with hypertensive disorder complicating pregnancy, 2 with gestational diabetes mellitus, 1 with pregnancy obesity, 1 with threatened premature birth and 2 who were lost to follow-up). Finally, there were 30 pregnant women who ultimately delivered at term. Among them, 25 pregnant women underwent spontaneous delivery, and 5 pregnant women underwent cesarean section. To ensure the consistency of the research results and reduce the impact of confounding factors, in this study, 25 pregnant women who underwent spontaneous delivery were enrolled. The placental specimens were collected from all pregnant women who ultimately delivered at term. The inclusion criteria for this study were age less than 35 years old, singleton pregnancy and nulliparous. Gastrointestinal diseases, antibiotic use during pregnancy, history of hypertension and diabetes, hyperthyroidism, autoimmune diseases such as rheumatic diseases, history of endocrine and metabolic diseases, history of blood transfusion, organ transplantation or immunotherapy were the exclusion criteria. A technical flow chart for the screening of research subjects is provided in Fig. 1.

### Sample collection

Placental specimens were collected under clean and sterile conditions within 30 min of spontaneous delivery at term. The placental surfaces were excised and discarded, and the area of necrosis and calcification of the maternal surface of the term placenta were avoided. On the fetal side of the placenta, four 1*1*1 cm cuboidal sections are circumferentially excised from separate areas of the placenta, each located 3 cm from the cord insertion site. Finally, placental specimens were immediately placed in a dedicated specimen box, transported to the laboratory within half an hour, and then stored at − 80 °C until DNA extraction.

### Collection of the clinical metadata of participants

After obtaining patient consent, we collected clinical metadata including age, height, weight before delivery, BMI before delivery, Apgar score, birth weight, head circumference and length of the newborns in the electronic database of the First Affiliated Hospital of Jinan University (Table 1).

**Table 1:** General clinical metadata of subjects in this study

Clinical metadata	Control Group (*n* = 15)	Probiotics Group (*n* = 10)	*P value*
Age (years)	27.7 ± 1.10	27.2 ± 1.16	0.763
Height (cm)	162.5 ± 2.12	158.6 ± 1.15	0.171
Weight before delivery (kg)	65.5 ± 2.27	65.3 ± 2.86	0.958
BMI before delivery (kg/m^2^)	24.8 ± 0.57	25.9 ± 0.98	0.286
1-min Apgar score	9	9	
5-min Apgar score	10	10	
Birth weight of newborn (kg)	3.5 ± 0.18	3.3 ± 0.17	0.671
Birth length of newborn (cm)	49.7 ± 0.41	50.1 ± 0.51	0.595
Head circumference of newborn (cm)	35.6 ± 1.85	33.9 ± 0.30	0.480

#### Research methods

##### Protein preparation and Western blot analysis

Total protein was extracted, the supernatant was collected, and a BCA protein assay kit (AR0146 Beijing Pulilai Gene Technology Co., Ltd.) was used for protein quantification. The placental autophagy-related proteins were separated by 12% sodium dodecyl sulfate polyacrylamide electrophoresis (SDS-PAGE) and transferred to a polyvinylidene difluoride (PVDF) membrane (Millipore). The membranes were incubated in blocking buffer containing 5% skimmed milk powder at room temperature for 1 to 2 h and at 4 °C overnight. The membranes were then incubated with primary rabbit antibodies against LC3B at a 1:500 dilution, Beclin1 at 1:1000 and GAPDH at a 1:1000 dilution (NB-Ab48394, NB-Ab217179 and NB-Ab181602, respectively, Abcam) at 4 °C overnight. The membrane was washed with Tris-buffered saline and Tween buffer solution six times and then incubated with a goat anti-rabbit secondary antibody at 1:8000 (NB-BA1054, BOSTER) for 2 h. Membranes were developed with ECL (BOSTER). The relative gray value of each band was analyzed using Quantity One software (LG2000, Hangzhou Langji Scientific Instrument Co., Ltd.), and then the relative expression of LC3 and Beclin1 proteins was calculated.

##### RNA extraction and real-time quantitative polymerase chain reaction

Total RNA from the placental tissue was extracted by TRIzol reagent (Invitrogen) according to the manufacturer’s instructions and stored at − 20 °C. A total of 4 μl of RNA template was used for reverse transcription to complementary DNA using multiscribe reverse transcriptase at 37 °C for 60 min, followed by 95 °C for 3 min. The reverse transcription reaction system included RNase Free d H_2_O 23.4 μl, 5 × RT Buffer 8 μl, C-MMLV 1 μl, oligo dT primers (50 μM) 1.2 μl, Random 6 mers (100 μM) 1.2 μl, d NTP 1.2 μl and RNA 4 μl. All primers were designed according to the ABI 3900 benchtop high-throughput DNA synthesizer instructions by Sangon Biotech (shanghai) Co., Ltd. (Table 2). The expression level of the autophagy-related genes was normalized to the levels of the housekeeping gene β-actin, which was used as an endogenous reference. The amplification cycles consisted of: 95 °C for 3 min, followed by 40 cycles of 95 °C for 10 s (denaturation) and 60 °C for 30 s (annealing), followed by 72 °C for 5 min (extension). The reaction was carried out in a C1000 Thermal cycler type real-time PCR machine. The RT-PCR mix included 7 μl of H2O, 10 μl of SYBRTM Premix Dimer Eraser (2×), 0.5 μl of forward primer (10 pmol/μl), 0.5 μl of reverse primer (10 pmol/μl) and 2 μl of cDNA.

**Table 2:** Primer sequences and amplified fragment list

Name	Forward primer	Reverse primer	Fragment size
LC3	5′-GAGAGCAGCATCCAACCAAA-3′	5′-ACATGGTCAGGTACAAGGAAC-3′	103 bp
Beclin1	5′-CGAGGGATGGAAGGGTCTAAG-3′	5′- GTTCCTGGATGGTGACACGG-3′	130 bp
β-actin	5′- GCATGGGTCAGAAGGATTCCT-3′	5′- TCGTCCCAGTTGGTGACGAT −3′	106 bp

#### Statistical analysis

Clinical metadata analysis was performed using SPSS version 19.0 (SPSS Inc., Chicago, IL, USA). where continuous data for the study subjects are presented as the mean ± SD. Two independent sample t-tests were used to compare the means. The chi-square test was used for the comparison of categorical data. In all analyses, *P* < 0.05 was considered statistically significant.

## Results

### Clinical characteristics of the study subjects

For this study, the baseline characteristics of the pregnant women who underwent had a term delivery are presented in Table 2. Altogether, 25 pregnant women were included. There were no significant differences in the clinical metadata between two groups (all *P* > 0.05, Table 1).

### Western blotting analysis of the expression levels of the autophagy-related proteins LC3 and Beclin1

**Fig. 2:**
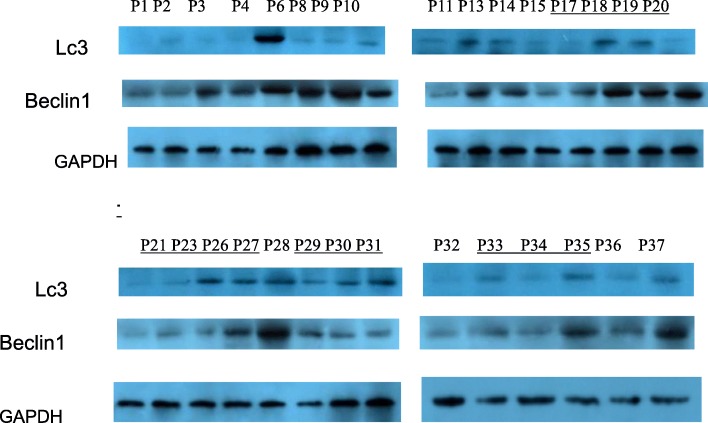
representative western blot for LC3 and Beclin1 on term placenta of two groups. Note: Control Group:P1-P15、P28、P32、P36、P37 Probiotic Group:P17-P27、P29-P31、P33-P35

**Fig. 3:**
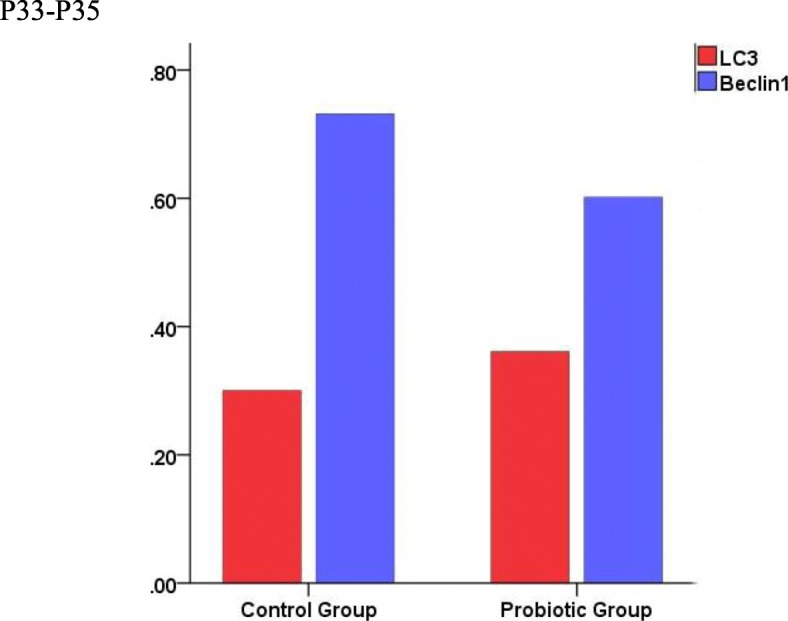
The analysis of data from optical density values of Western blotting bands of two groups. Note: “*” means the difference is statistically significant (*p* < 0.05)

Figure 2 shows representative Western blots for LC3 and Beclin1 in the term placentas of the two groups. As displayed in Fig. 3, there was no significant difference in either LC3 or Beclin1 between the control group and the probiotic group(*P* > 0.05).

### Rt-PCR (reverse transcription-polymerase chain reaction) analysis of the content of LC3 mRNA and Beclin1 mRNA

**Fig. 4:**
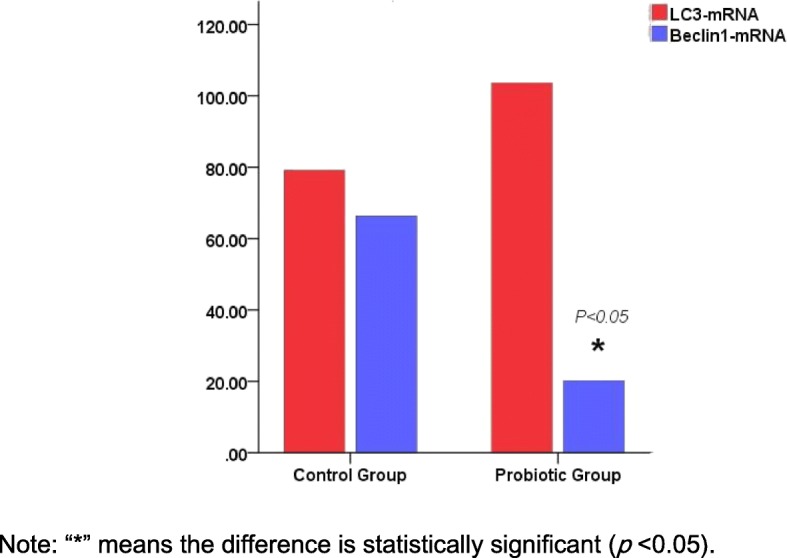
Comparison of autophagy-related proteins LC3-mRNA and Beclin1-mRNA on term placenta of two groups. **a**: LC3 protein amplification curve (**b**): LC3 protein melt curve (**c**): Beclin1 protein amplification curve (**d**): Beclin1 protein melt curve

**Fig. 5:**
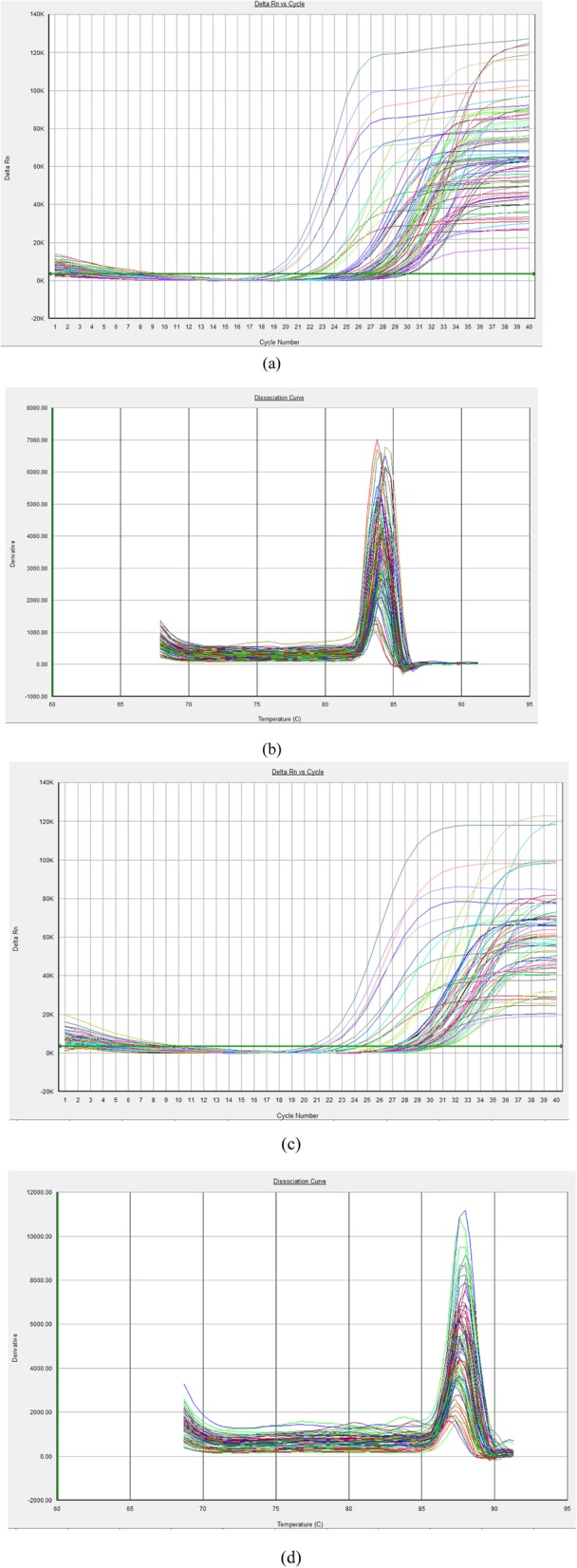
Rt-PCR amplification curve and melt curve of LC3 and Beclin1 mRNA on term placenta of two groups

As shown in Fig. 4, compared with that in the placentas from the control group, the Beclin1 mRNA, but not the LC3 mRNA, was decreased in the placentas from the probiotic group (*P* < 0.05). The basal expression level of LC3 mRNA was slightly, although not significantly, lower than that in the corresponding control (Fig. 4); however, the basal expression level of Beclin1 mRNA was significantly lower than that in the control. These findings indicate that the probiotic supplement had a controversial effect on the expression of genes involved in placental autophagy, but the overall effect was suppressive. Figure 5 represents the RT-PCR amplification curve and melt curve of LC3 and Beclin1 mRNA in the term placentas of the two groups.

## Discussion

Autophagy plays an important role in the regulation of placental function and the maintenance of normal pregnancy by participating in eukaryotic cell self-metabolism and in the establishment of utero-placental circulation [10]. Our results indicate that the supplementation of probiotics can impose a slight but not significant influence on placental autophagy, and paradoxically, the influence is controversial. At the protein level, there was increased expression of LC3, but the expression of Beclin1 was decreased (Fig. 3). Moreover, no significant difference was found in LC3 mRNA between the two groups (Fig. 4), whereas the Beclin1 mRNA expression in the probiotic group was significantly lower than that in the control group (*P* < 0.05, Fig. 4). The reason underlying the lack of significance may be that the two methods are focus on the mRNA level and protein level (i.e., transcription and translation, respectively), where there is posttranscriptional processing after transcription, degradation of the transcription products, translation, posttranslational processing and modification. The sensitivity of RT-PCR for detecting mRNA is very high, so there may be discrepancies in the detection results of the above two methods. Moreover, epigenetic mechanisms such as DNA methylation may also play an important role in the results. Thus, orally supplemented probiotics might decrease the levels of the autophagy-related protein Beclin1 mRNA in the placenta.

However, the autophagy phenomenon of placental trophoblast cells is affected by various factors, such as delivery mode, nutrient supply and pregnancy pathology. Recently, it has been reported that the mode of delivery directly affects placental oxidative stress and autophagy [20]. Since normal pregnancy in spontaneous vaginal delivery can intermittently affect the blood flow of the placenta and mechanical stress caused by muscle contraction, placental autophagy activity may be enhanced. However, Signorelli et al. showed that placental autophagy activity was significantly reduced compared with that of cesarean section [21]. Furthermore, Doulaveris reported that the inhibition of autophagy is associated with spontaneous vaginal delivery, and may be caused by preoperative fasting and the use of anesthetic drugs [22]. Some scholars have shown that the use of local anesthetics in rabbits can promote the activation of the autophagy signaling pathway, thereby enhancing autophagy [23]. Preoperative fasting will lower the levels of glucose and amino acids and increase the level of autophagy to ensure energy supply and normal metabolism [24].

Studies have shown that the expression of the autophagy proteins LC3 and Beclin1 in pregnant women with preeclampsia is higher than that in normal pregnant women [11]. However, some scholars have not found significant differences between preeclamptic patients and normal pregnant women by comparing the placental autophagy-related pathway genes [25]. Moreover, What’s more, by collecting the placental tissue of 61 pregnant women who underwent elective cesarean section, Hung T et al. found that the expression of autophagy-related proteins LC3 and Beclin1 in the placental tissues of pregnant women with intrauterine growth restriction was higher than that of normal pregnant women [26]. The pathological process of preeclampsia is accompanied by impaired functioning of the placental trophoblast cells, insufficient remodeling of the uterine spiral arteries, and oxidative damage. Fetal growth restriction may also have pathological processes causing placental dysfunction. It is therefore possible that placental autophagy activity is increased to further help trophoblasts adapt to these pathological changes to maintain bioenergy homeostasis and clear damaged organelles. Pregnant women with pathological pregnancy will have increased expression of autophagy-associated proteins in the placenta. Moreover, although we strove to maintain the homogeneity of the subjects, there were still some potential confounding factors that could interfere with the results, such as food consumption, alcohol consumption, and physical activity. Studies have shown that there is a special “channel” for oral supplementation. Accumulating evidence suggests that supplementation with probiotics can induce immune modulation and thus prevent certain intestinal diseases [27, 28]. Moreover, Wu et al. showed through Western blot and confocal laser scanning analysis [29] that the probiotic *Bacillus amyloliquefaciens* SC06 induces autophagy to protect against pathogens in macrophages in a dose- and time-dependent manner. Moreover, Lin et al. reported that Bifidobacterium can initiate autophagy activation in intestinal epithelial cells [19]. Although the specific mechanism is unknown, probiotics can regulate autophagy in the placentas of normal pregnant women. This may have a positive effect on the fetus and infant or provide a clinical basis for probiotic use in pathological pregnancy, which needs to be further explored.

To our knowledge, the study has strengths. This is the first study investigating the influence of oral probiotic supplementation on placental autophagy. The placental tissue included in this study was obtained from normal spontaneous delivery, excluding differences caused by cesarean section. However, immunofluorescence staining was not performed, without which we could not better evaluate the microscopic difference of the placenta after supplementation with probiotics.

## Conclusions

Overall, orally supplemented probiotic at 32 weeks of gestation was found to affect the autophagy protein Beclin1 in placentas undergoing spontaneous delivery during normal pregnancy. This may provide new insight for probiotic-mediated placental protection. Findings from this study indicate that probiotic supplementation may induce a reduction in the autophagy-related protein Beclin1 at the mRNA level in the placentas undergoing spontaneous delivery. Based on this result, we speculate that the clinical supplementation of normal pregnant women with probiotics may prevent the occurrence of placenta-derived diseases, but the evaluation of specific clinical effects still requires multisample randomized-controlled trial (RCT) experiments for confirmation.

## Data Availability

The datasets generated and/or analyzed during the current study are not publicly available but are available from the corresponding author on reasonable request.

## Abbreviations

ISAPP: International Scientific Association for Probiotics and Prebiotics
LC3: microtubule associated protein l light chain3
SDS-PAGE: Sodium dodecyl sulfate polyacrylamide gel electrophoresis
PVDF: Polyvinylidene difluoride
SPSS: Statistical Package for Social Sciences
Rt-PCR: Reverse Transcription-Polymerase Chain Reaction
IRB: Institutional Review Board
